# Supplementary material for: Saccharomyces boulardii (CNCM I-745) improves intestinal damage in sepsis by remodeling intestinal flora structure
Source: Front Cell Infect Microbiol. 2025 Jul 31;15:1602792. doi: 10.3389/fcimb.2025.1602792 (PMC12350329; doi:10.3389/fcimb.2025.1602792)
Supplement: Supplementary file 1 [file DataSheet1.zip › summary/summary/7_Differential_analysis/1_diff_abundance/5_Family/Group.DW4_vs_DW0_vs_CW4_vs_CW0_vs_BW4_vs_BW0_vs_AW4_vs_AW0/Group.DW4_vs_DW0_vs_CW4_vs_CW0_vs_BW4_vs_BW0_vs_AW4_vs_AW0_diff_barplot.pdf]

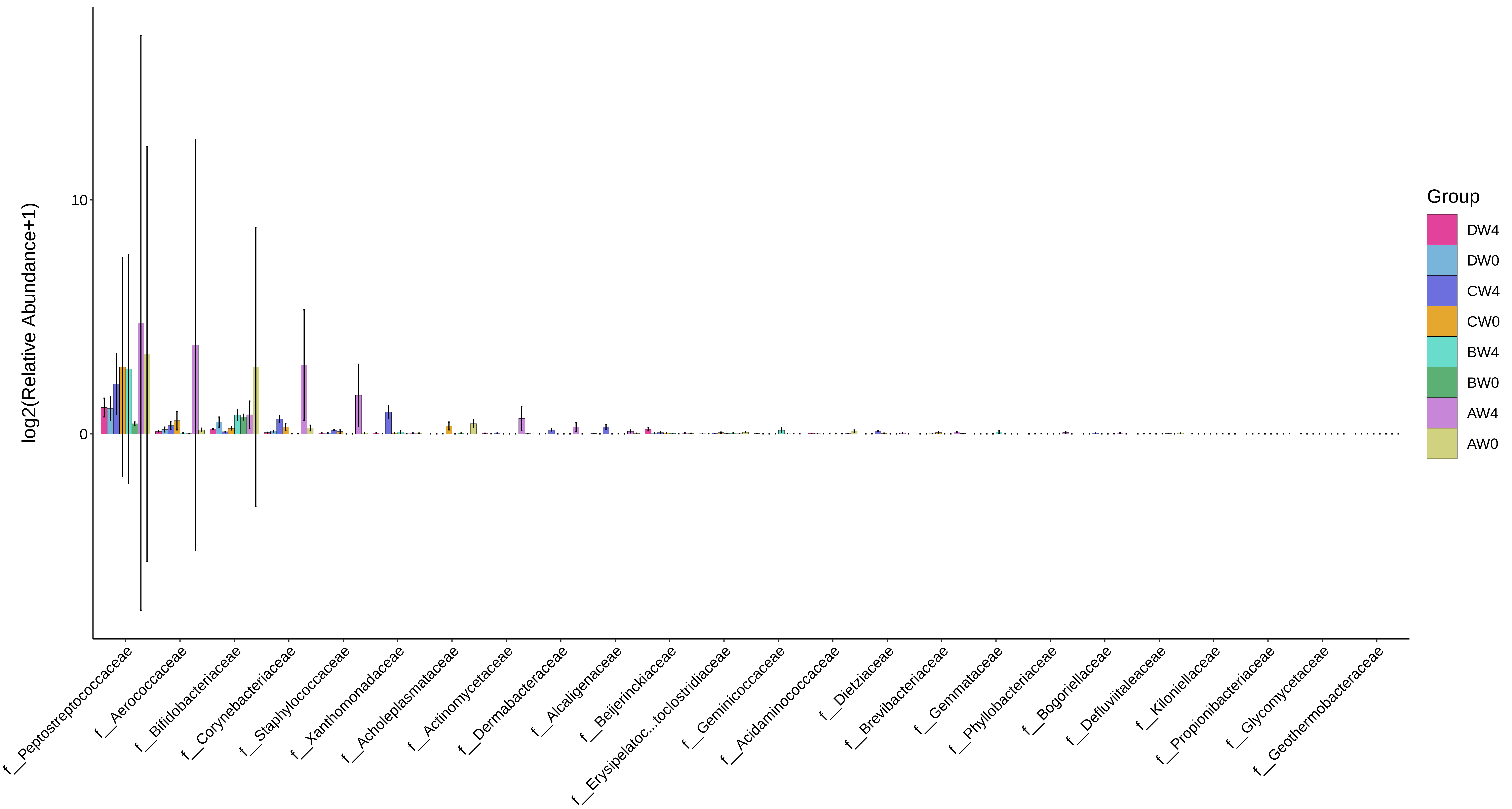

log2(Relative Abundance+1)
10
0
f__Peptostreptococcaceae
f__Aerococcaceae
f__Bifidobacteriaceae
f__Corynebacteriaceae
f__Staphylococcaceae
f__Xanthomonadaceae
f__Acholeplasmataceae
f__Actinomycetaceae
f__Dermabacteraceae
f__Alcaligenaceae
f__Beijerinckiaceae
f__Erysipelatoc...toclostridiaceae
f__Geminicoccaceae
f__Acidaminococcaceae
f__Dietziaceae
f__Brevibacteriaceae
f__Gemmataceae
f__Phyllobacteriaceae
f__Bogoriellaceae
f__Defluviitaleaceae
f__Kiloniellaceae
f__Propionibacteriaceae
f__Glycomycetaceae
f__Geothermobacteraceae
Group
DW4
DW0
CW4
CW0
BW4
BW0
AW4
AW0
